# Olfactory and taste disorders in COVID-19: a cross-sectional study in primary health care

**DOI:** 10.1590/1806-9282.20231018

**Published:** 2024-01-22

**Authors:** Assel Muratovna Shigayeva Ferreira, João Agnaldo do Nascimento, Letícia de Carvalho Palhano Travassos, Leandro de Araújo Pernambuco

**Affiliations:** 1 Universidade Federal da Paraíba – João Pessoa (PB), Brazil.

**Keywords:** COVID-19, Olfaction disorders, Taste disorders, Primary care

## Abstract

**OBJECTIVE:**

The objective of this study was to describe the occurrence of self-reported olfactory and taste disorders in non-hospitalized Brazilian adults who presented severe acute respiratory syndrome-related coronavirus 2 infection symptoms and attended primary health care.

**METHODS:**

This cross-sectional study was based on a routine standardized diagnostic screening questionnaire applied in a Brazilian primary care facility. The olfactory and taste disorder occurrence was compared between severe acute respiratory syndrome-related coronavirus 2-positive and severe acute respiratory syndrome-related coronavirus 2-negative cases and described by age and sex.

**RESULTS:**

Severe acute respiratory syndrome-related coronavirus 2-positive patients had a higher proportion of self-reported olfactory and taste disorders, as compared with severe acute respiratory syndrome-negative (50.7%, vs. 20.6%, p<0.0001). Of all individuals with self-reported olfactory and taste disorder cases, 69% presented both olfactory and taste impairments, 13% olfactory only, and 17% taste only. In severe acute respiratory syndrome-related coronavirus 2-positive cases, the frequency of olfactory and taste disorders was significantly higher among females as compared with males (71% vs. 34%). Additionally, people with olfactory and taste disorders were significantly younger in the severe acute respiratory syndrome-related coronavirus 2-positive group.

**CONCLUSION:**

Self-reported olfactory and taste disorders are highly common among non-hospitalized severe acute respiratory syndrome-related coronavirus 2-positive Brazilian people who attended the Family Health Care Unit. The co-occurrence of both self-reported olfactory and taste disorders was more frequent than self-reported olfactory or taste disorders alone.

## INTRODUCTION

Early diagnosis of viral infection caused by SARS-CoV-2 is essential for the management of associated illness, referred to as COVID-19^1^. The clinical manifestation of this disease varies broadly from mild, flu-like conditions to life-threatening acute respiratory syndrome. Thus, the understanding of the predictive value of symptoms caused by SARS-CoV-2 infection is important for timely diagnosis and effective health care.

Besides non-specific flu-like and respiratory symptoms, sudden loss of smell (hyposmia/anosmia) and/or taste (hypogeusia/ageusia) emerged as one of the distinct features of COVID-19 onset or early stages of illness, being often the only reported symptoms^2-8^. Although olfactory and taste disorders (OTDs) inducted by SARS-CoV-2 received most of the attention in the early period of the COVID-19 pandemic, several recent studies suggested that OTDs may remain a diagnostic marker of suspicion of SARS-CoV-2 infection^9,10^. The occurrence of olfactory and taste impairment among COVID-19-positive patients is well acknowledged, although it could change over time and settings^5-11^. Therefore, despite many community-based studies observed for non-hospitalized patients, there are still limited data from primary care settings^3,5,7^.

Brazil was among the countries that were most affected by the COVID-19 pandemic. In 2020–2021, the country registered one of the highest numbers of cases and deaths in the world^12^. The limited access to virus testing during this period led to national health care systems relying on the isolation of mild cases who presented flu-like manifestations, including olfactory and taste symptoms. Nevertheless, less is known about the prevalence of OTDs among COVID-19 patients during the pandemic in Brazil regarding its regional differences, seroprevalence, health care settings, and other characteristics. Furthermore, only a few studies reported non-hospitalized OTD cases among COVID-19 patients who were enrolled via telemedicine^13^ or by online surveys^14^. In Brazil, primary health care as a part of the universal public Health Care System (Sistema Único de Saúde (SUS)) plays a key role in the implementation of certain policies for improving population health and promotion of health equity. While research supports the practical issues related to community health and its demands, it can contribute to the development of certain primary care preventive policies toward disease’s consequences or future outbreaks^15^.

Therefore, this study aimed to investigate the occurrence of OTDs among patients presenting with COVID-19 in a Brazilian primary care facility in the period between March and December 2021. We compare the frequency of OTDs between confirmed SARS-CoV-2-positive and -negative cases and describe the occurrence of OTDs by age and gender.

## METHODS

### Study design and settings

This is a cross-sectional study that used a standardized diagnostic questionnaire. The study was conducted in a Family Health Care Unit (Unidade de Saúde da Família – USF), in Joao Pessoa city city of Paraiba state, Brazil.

**Figure 1.**
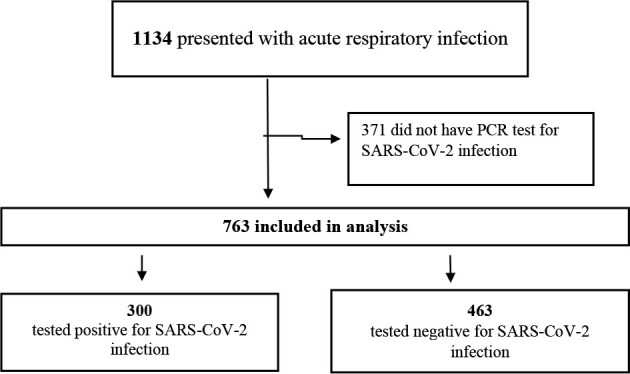
Flowchart of study sample selection.

We initially included all adult people, ≥18 years old, who were presented in a study setting with symptoms of acute respiratory infection (ARI) in the period between March 1, 2021, and December 31, 2021. During this period of pandemic, any individual with ARI symptoms was considered a suspect of SARS-CoV-2 infection^16^. Figure 1 presents a flowchart of selection and inclusion into the analysis.

All patients suspected of COVID-19 and who were presented at the Family Health Care Unit routinely received a standardized diagnostic questionnaire. The diagnostic questionnaire was part of a routine diagnostic procedure conducted by the outpatient clinic research team at the time of pandemic (2020–2021). The questionnaire asked respondents about the presence of active symptoms, including self-assessment of the patients’ current smell and taste function. Demographic data (e.g., age and sex), date of onset, list of the preexisting conditions, and date of SARS-CoV-2 test if performed were collected. For the analysis, 763 patients who had an RT-PCR test for SARS-CoV-2 infection were included.

We estimated and compared the frequency of self-reported OTDs among SARS-CoV-2-positive and SARS-CoV-2-negative patients, overall and by age and sex. OTDs were grouped as having reported: (i) olfactory, defined as reduction/change or loss of smell (hyposmia/anosmia), (ii) taste (hypogeusia/ageusia), defined as reduction or loss of taste, and (iii) both OTDs.

### Statistical methods

Descriptive analyses were performed for continuous and categorical variables. The sample was described by study groups, i.e., SARS-CoV-2-positive and SARS-CoV-2-negative. Patients’ demographic characteristics and frequency of OTDs were compared using the chi-square test and independent-samples t-test.

### Ethics considerations

The study was approved by the Brazilian Ethical Review Board, as required for conducting research involving humans (nº5.376.051; CAAE: 55764722.4.0000.5188).

## RESULTS

The characteristics of 763 participants included in the study groups are presented in Table 1. From this sample, 300 individuals tested positive for SARS-CoV-2, and 463 tested negative. The mean age was 41.6 (range 18–89) years in the SARS-CoV-2-positive group and 37.9 (range 18–80) years in the negative group (p<0.0001). We observed a higher proportion (56.3%) of men in the SARS-CoV-2-positive group as compared with the negative group (46.7%) (p<0.0001).

**Table 1.** Characteristics of patients and frequency of olfactory and taste disorders on initial presentation at a primary care clinic, by the severe acute respiratory syndrome-related coronavirus 2 test status.

	SARS-CoV-2-positive	SARS-CoV-2-negative	OR [95%CI]	p-value^*^
	n=300	n=463		
	n (%)	n (%)		
Sex, male	169 (56.3%)	216 (46.7%)	–	<0.0001
Age in years (Median, IQR)	40.5 (18–89)	37.0 (18–80)	–	
(Mean, SD)	41.6 (±13.8)	37.9 (±12.9)	–	<0.0001
OTDs, any	152 (50.7%)	96 (20.7%)	2.4 [1.98–3.01]	<0.0001
Olfactory (hyposmia/anosmia)	20 (6.7%)	20 (4.3%)	1.5 [0.84–2.81]	0.15
Taste (hypogeusia/ageusia)	27 (9.0%)	21 (4.5%)	2.0 [1.14–3.44]	0.01
Both OTDs	105 (35.0%)	55 (11.9%)	2.9 [2.20–3.94]	<0.0001

SARS-CoV-2: severe acute respiratory syndrome-related coronavirus 2; OTDs: olfactory and taste disorders; OR: odds ratio; CI: confidence interval; IQR: interquartile range; SD: standard deviation;

* chi-square test.

The overall prevalence of self-reported OTDs was higher in SARS-CoV-2-positive group (50.7%). The chance to have any self-reported OTDs was 2.4 higher in this group compared with the SARS-CoV-2-negative group (95%CI 1.98–3.01, p<0.0001). In the group with 152 SARS-CoV-2-positive patients with self-reported OTDs, 69.1% (105/152) presented with both OTDs, 13.2% (20/152) with olfactory only, and 17.7% (27/152) with taste only disorder. In the group with 96 SARS-CoV-2-negative participants with self-reported OTDs, 57% (55/96) had both disorders, 21% (20/96) reported only olfactory, and 22% (22/96) had only the taste loss symptoms. The chance of having both OTDs in the SARS-CoV-2-positive group was approximately three times higher than in patients who tested negative for the virus (OR=2.9 [2.20–3.94], p<0.0001).

The overall frequency of OTDs in SARS-CoV-2-positive participants was higher in females (71.8%) as compared with males (34.3%) (Table 2). In contrast, there was no difference in the occurrence of OTDs by sex in the SARS-CoV-2-negative group. People with OTDs were significantly younger in the SARS-CoV-2-positive group. However, in the SARS-CoV-2-negative group, age was not related to OTDs.

**Table 2.** Frequency of olfactory and taste disorders by sex and age among severe acute respiratory syndrome-related coronavirus 2-positive and -negative patients.

	SARS-CoV-2-positive				SARS-CoV-2-negative			
	n=300				n=463			
	OTDs		p-value	OR [95%CI]	OTDs		p-value	OR [95%CI]
	Yes	No			Yes	No		
Sex, n (%)								
Male	58 (34.3)	111 (65.7)	<0.001^*^	0.20 [0.12–0.33]	41 (19.0)	175 (81.0)	0.38	–
Female	94 (71.8)	37 (28.2)			55 (22.3)	192 (77.7)		
Age								
Mean (±SD)	39.66 (±13.9)	44.44 (±14.1)	0.003^**^	–	41.18 (±13.4)	38.25 (±14.3)	0.07	–

SARS-CoV-2: severe acute respiratory syndrome-related coronavirus 2; OTDs: olfactory and taste disorders; OR: odds ratio; CI: confidence interval; SD: standard deviation;

* chi-square test;

** independent-samples t-test.

## DISCUSSION

The occurrence of OTDs in COVID-19 patients, isolated or combined, varies dramatically across populations^8-10^. However, the knowledge of OTD occurrence among COVID-19 patients in Brazil regarding its regional differences and characteristics of patients who attended on the ambulatory level during the pandemic is still limited, especially in the family health care units.

Our results support previous findings that olfactory and gustatory dysfunctions are both prevalent in patients with mild COVID-19 infection and that they typically present as a combined disorder^8,9,17^. The underlying mechanism of COVID-related OTDs remains a topic of investigation: the contribution of local inflammation caused by the virus and its invasion to olfaction epithelium via the angiotensin-converting enzyme-2 (ACE-2)^18^, central olfaction pathway, interconnection of retro-nasal olfaction to taste perception, and direct damage of taste function caused by the presence of virus^9^ are some of the most cited evidence.

Regarding sex distribution, the higher occurrence of olfactory and taste symptoms among females was reported previously^8,19^, yet the potential sex differences in the development of symptoms remain unclear^20^.

Olfactory and taste impairments were found to be highly common among adults with positive SARS-CoV-2 infection and they are generally younger than those who tested negative. Data based on ambulatory settings observations^19,21^ reported a higher prevalence of OTDs in young patients with COVID-19, who generally had mild or moderate form of infection, in contrast to a higher prevalence of OTDs in older patients with severe hospitalized cases^22^. While the mechanisms of OTD development in SARS-CoV-2-positive patients remain unclear^23^, the loss of taste and smell in aging non-COVID patients is widely described in scientific literature^24,25^.

The study has limitations: it refers to a community subset that self-reported OTD symptoms without observation of symptom development or objective screening. The patients who tested negative for SARS-CoV-2 but had flu-like symptoms were not tested for other types of infections (e.g., influenza). The study relied on RT-PCR test results only.

Our findings contribute to a better understanding of OTD occurrence among COVID-19 patients in Brazil who attended primary health care setting. Therefore, further studies are needed to investigate the prevalence of OTDs among COVID-19 patients.

## CONCLUSION

Self-reported OTDs are highly common among non-hospitalized SARS-CoV-2-positive Brazilian people who attended primary health care. The co-occurrence of both self-reported OTDs was more frequent than self-reported olfactory or taste disorders alone.
